# Applicability of Longitudinal Strain of Left Ventricle in Unstable Angina

**DOI:** 10.5935/abc.20180062

**Published:** 2018-04

**Authors:** Natasha Soares Simões dos Santos, Andrea de Andrade Vilela, Rodrigo Bellio de Mattos Barretto, Marcela Paganelli do Vale, Mariana Oliveira Rezende, Murilo Castro Ferreira, Alexandre José Aguiar Andrade, Nelson Henrique Goes Scorsioni, Olívia Ximenes de Queiroga, David Le Bihan

**Affiliations:** Instituto Dante Pazzanese de Cardiologia, São Paulo, SP - Brazil

**Keywords:** Angina, unstable / physiopathology, Ventricular Dysfunction, Left, Myocardial Ischemia / physiopathology, Strain, Echocardiography / methods

## Abstract

**Background:**

Unstable angina (UA) is a common cause of hospital admission; risk stratification helps determine strategies for treatment.

**Objective:**

To determine the applicability of two-dimensional longitudinal strain (SL2D) for the identification of myocardial ischemia in patients with UA.

**Methods:**

Cross-sectional, descriptive, observational study lasting 60 days. The sample consisted of 78 patients, of which fifteen (19.2%) were eligible for longitudinal strain analysis. The value of p < 0.05 was considered significant.

**Results:**

The group of ineligible patients presented: a lower proportion of women, a higher prevalence of diabetes mellitus (DM), use of ASA, statins and beta-blockers and larger cavity diameters. The main causes of non-applicability were: presence of previous infarction (56.4%), previous CTA (22.1%), previous MRI (11.5%) or both (16.7%) and the presence of specific electrocardiographic abnormalities (12.8%). SL2D assessment revealed a lower global strain value in those with stenosis greater than 70% in some epicardial coronary arteries (17.1 [3.1] versus 20.2 [6.7], with p = 0.014). Segmental strain assessment showed an association between severe CX and RD lesions with longitudinal strain reduction of lateral and inferior walls basal segments; (14 [5] versus 21 [10], with p = 0.04) and (12.5 [6] versus 19 [8], respectively).

**Conclusion:**

There was very low SL2D applicability to assess ischemia in the studied population. However, the global strain showed a correlation with the presence of significant coronary lesion, which could be included in the UA diagnostic arsenal in the future.

## Introduction

In United States, unstable angina (UA) is the most common cardiovascular cause of hospitalization and is responsible for most hospitalizations in coronary units.^1^ The diagnosis of UA is performed by clinical criteria based on angina duration and intensity.^2^ UA patient has a variable prognosis for unfavorable events such as acute myocardial infarction (AMI), recurrence of angina, necrosis biomarkers, ventricular function and need for myocardial revascularization.^3^

Speckle tracking (ST) is a technology introduced in the 1980s that allows the quantification of global and regional myocardial deformity by tracking the natural heart muscle "acoustic marks" by ultrasound, presenting reduced values in presence of myocardial ischemia.^4,5^ ST allows myocardial strain calculation and has shown great utility in the identification of subendocardial ischemia as in unstable angina, with greater sensitivity and specificity than two-dimensional echocardiogram.^6^

However, for ST to track adequately speckles, there are some variables that may interfere in deformity analysis, so when present, they may give erroneous results or even impede myocardial strain analysis. In addition, for myocardial ischemia identification in UA patients, infarction previous myocardial presence or other myocardial injury (such as significant valvar heart disease) may alter myocardial deformity and cause an incorrect analysis of deformity decrease true cause. These are the variables that interfere in myocardial deformity correct analysis, and for that reason, they are considered exclusion criteria in the majority of published studies (aimed at analyzing acute ischemia): previous infarction, atrial fibrillation, left bundle branch block, ventricular arrhythmia aortic and/or mitral valvar disease, previous cardiac surgery, ventricular hypertrophy, cardiac pacemaker and inadequate acoustic window.^7^

The main objective is to study the applicability prevalence of two-dimensional longitudinal strain (SL2D) in all hospitalized patients diagnosed with UA during the 60-day observation period.

The secondary objective is to evaluate the diagnostic capacity of SL2D in the identification of culprit vessel due to ischemic event in UA patients.

## Methods

This is a cross-sectional, descriptive, performed at the Emergency Room (ER) and Coronary Unit (CU) of Dante Pazzanese Institute of Cardiology (IDPC).

Inclusion criteria were: hospitalized patients of both sexes, age greater than 18 years and clinical diagnosis of UA who were admitted to IDPC service during the study period and who accepted the participation in the study, having signed informed consent form. We emphasize that there was no calculation of sample size. A census was carried out of all patients who had inclusion criteria. Patient arrival to IDPC service was by convenience.

Exclusion criterion was the change in diagnosis during hospitalization. These cases occurred in patients who entered the service with initial chest pain and after propaedeutic and complementary exams, diagnosis of UA was ruled out. In case of differential diagnosis and closing as final diagnosis: acute myocardial infarction (AMI) with supra or no supra-ST segment, aortic dissection, pulmonary embolism and aortic stenosis.

We analyzed clinical-epidemiological and electrocardiographic characteristics, as well as tests collection for troponin I and creatinine.

Risk stratification was done using GRACE risk score.^8^^.^^9^

Electrocardiographic analysis was performed by two experienced cardiologists; in case of disagreement regarding the diagnosis, tracing would be analyzed by a specialized service in the electrocardiographic reports of the institution where the research was carried out. Transthoracic echocardiography was performed within 48 hours of patient precordial pain last episode in ER or CU. The equipment for conducting examination was GE® Vivid E9 (General Electric Medical System, Norway) with transducer "array in phase" with 3.5 megahertz emission frequency. Images obtained during the examination were acquired with harmonic, in a repetition of frames between 50 and 70 frames/second, in digital clips form ( three consecutive cycles average) and recorded in CDs for later analysis in workstation EchoPAC PC version 6.0.1® (GE VingmedUltrasound).

According to American Society of Echocardiography and European Society of Echocardiography committee guidelines, which standardized the acquisition of tomographic sections obtained during echocardiographic examinations, with the patient in left lateral decubitus and electrocardiogram monitored, we acquired transthoracicly echocardiographic images by the Spectral Doppler (pulsatile and continuous) Doppler and flow mapping in color.^10^

Measures acquired:

- Two-dimensional: diastolic and systolic left ventricular (LV) diameter, left atrium anteroposterior diameter (LA), aortic root diameter, interventricular septum and posterior wall thickness. LV diastolic and systolic volume. Calculation of LV ejection fraction (EF) by the modified biplanar Simpson method.
- Doppler and Color Flow Mapping: Mitral flow with spectral Doppler (pulsatile and continuous) for diastolic function analysis and mitral valvopathy quantification, when present. Aortic flow with spectral Doppler (pulsatile and continuous), to determine aortic valve opening and closing (to mark the systolic event), and aortic valvopathy quantification, when present. Valve lesions diagnosis and quantification followed American Society of Echocardiography recommendations.^11^

The technique to obtain longitudinal tension was done as follows:

- Marking systolic event with aortic flow pulsating Doppler.
- Determination of three points of endocardial border in each of the following images: apical 3 chambers (at anterosseptal wall base, at inferolateral wall base and at apex), apical 4 chambers (at septum base, at lateral wall base and at apex) and apical 2 chambers (at inferior wall base, at anterior wall base and at apex).
- Through the Automatic Function Imaging® (AFI) tool, the deformation of each of the 17 myocardial segments was automatically calculated, providing posteriorly left ventricle global deformation (analyzed segments mean). The program provides SL2D curves and polar map with longitudinal strain values in each segment.

**Figure 1:**
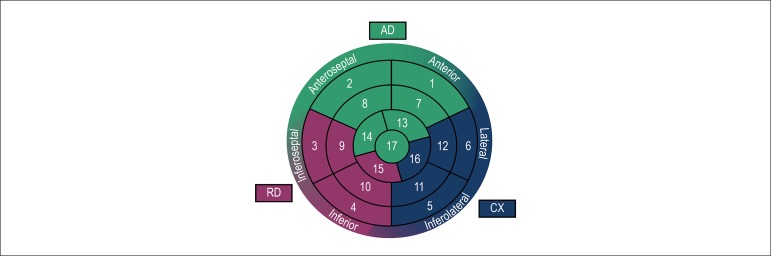
Polar map with coronary irrigation correlation. AD: anterior descending coronary artery; CX: circumflex coronary artery; RD: right coronary artery.

The maximum absolute value of the two-dimensional strain curve was defined as the systolic peak. Adjacent myocardial segments with altered strain value were defined as ischemic territory, correlating them with coronary irrigation, according to polar map shown in Figure 1.

According to the literature,^7,12,13^ the situations mentioned below may lead to a change in myocardial deformity, or to a real deformity impairment, or by limitation of the software to identify acoustic marks during the cardiac cycle:

- Concentric ventricular hypertrophy (LVH);
- Aortic and/or mitral valvar diseases greater than moderate degree;
- Pacemaker pace;
- At least one of the following electrocardiographic changes: left bundle branch block (LBBB), atrial fibrillation (AF) rhythm and complex ventricular arrhythmia;
- Secondary unstable angina (acute anemia, tachyarrhythmia and infection);
- Prior AMI or prior myocardial (percutaneous or surgical) revascularization procedure and
- Inadequate acoustic window.

Based on the above, we hypothesized that the presence of one of these alterations may impair SL2D analysis in severe coronary disease identification in UA patients. These concepts has fundamental importance for the knowledge of SL2D real applicability in this population, when the examination purpose is to evaluate coronary disease responsible for the acute condition.

Patients eligible for bidimensional echocardiogram with longitudinal strain were submitted to the method by two skilled and experienced professionals, who did not have access to information on patient coronary anatomy evaluated until the conclusion of the study.

Results of cardiac catheterization (CC) and Coronary Angiography by Computed Tomography (CACT) exams were also analyzed. Stenosis greater than or equal to 70% in epicardial coronary arteries or stenosis greater than or equal to 50% in left main coronary artery (LMCA) was considered.

The sample was divided into two groups: Group A - patients in whom it was possible to analyze by SL2D and Group B - patients in whom analysis by SL2D was not possible.

The research protocol was submitted and approved by the institution's Ethics and Research Committee.

There was no interference in individual medical conduct due to participation in the study. Such conduct was based on ER and CU routine that corresponds to US and national guidelines^3,14^ for UA patients treatment patients.

### Statistical analysis

Statistical analysis was performed with Statistical Package for Social Sciences (SPSS), version 19.0.

Kolmogorov-Smirnov and Shapiro-Wilk tests were performed to verify our sample normal distribution. As the normality hypothesis was rejected, we used nonparametric tests for analysis.

Groups were compared using Mann-Whitney test and Fisher exact test as appropriate.

Continuous variables were presented as median and interquartile range, and categorical variables were expressed as percentage (%).

ROC curve was used to evaluate SL2D discriminative power in severe coronary stenosis identification (≥ 70%) in UA patients.

Level of significance was 5%.

## Results

We evaluated 93 patients diagnosed with UA at admission to ER; however, fifteen (16.2%) patients were excluded from the study due to diagnosis change during hospitalization, 13 (14%) cases with non-ST-segment AMI, one (1.1%) with UA post-MI and one (1.1%) with type A aortic dissection.

At the end, 78 UA patients were investigated, of which fifteen (19.2%) were eligible for longitudinal strain analysis.

Main population clinical characteristics are summarized in Table 1.

**Table 1:** Clinical characteristics of studied population (n = 78)

	Median [p25- 75]
Age (years)	61,5 [53 - 69]
**Gender (%)**	
Male	60,3%
Female	39,4%
BMI (Kg/m^2^)	28,16 [24,47 - 30,71]
SBP (mmHg)	137 [122,75 - 154,25]
HR (bpm)	74 [69 - 83,5]
Serum Creatinine (mg/dL)	0,9 [0,7 - 1,1]
GRACE (points)	95 [81 - 117]
Troponin (pg/mL)	0,02 [0,01 - 0,05]
High blood pressure (%)	88,5%
Diabetes (%)	38,5%
Smoking (%)	32,1%
Dyslipidemia (%)	65,4%
Family history for CAD (%)	19,2%
Known CAD (%)	66,7%
**Medications in use (%)**	
ACEI	32,1%
ARB	41%
Beta blocker	65,4%
Acetylsalicylic acid	82,1%
Other antiplatelet	29,5%
Calcium channel blocker	33,3%
Statin	76,9%
Nitrate	37,2%
**Prior Intervention (%)**	
Surgical revascularization	11,5%
Angioplasty	22,1%
Angioplasty + Surgical revascularization	16,7%
Previous MI (%)	56,4%

BMI: body mass index; SBP: systolic blood pressure; HR: heart rate; CAD: coronary artery disease; AMI: acute myocardial infarction; ACEI: angiotensinogen converting enzyme inhibitor; ARB: angiotensin receptor AT-2 blocker.

About 70% of sample had no change in QRS complex duration or morphology complex; more than half (60.3%) showed no change in ventricular repolarization. Five patients (6.4%) presented ST segment depression on admission. Main electrocardiographic changes are detailed in Table 2.

**Table 2:** Electrocardiographic findings (n = 78)

Change	Frequency (%)
LBBCD	10,3%
RBBB	3,8%
LASDB	2,6%
RBBB + LASDB	2,6%
LBBB	3,8%
Q wave pathological	3,8%
Pacemaker pace	3,8%
High response AF	1,3%
CVR anterosseptal	5,1%
Previous CVR	5,1%
Lower CVR	9%
Side CVR	7,7%
Diffuse CVR	11,5%
Infra/ST > 0,5 mm	1,3%

LBBCD: left bundle branch conduction disorder; RBBB: right bundle branch block; LASDB: left anterior superior divisional block; CVR: change in ventricular repolarization; LBBB: left bundle branch block; AF: atrial fibrillation.

Of the 63 patients in whom the longitudinal strain was not applied, 40 (63.5%) performed two-dimensional echocardiography during ER stay. Main echocardiographic findings of this population, including the 15 patients submitted to SL2D, are shown in Table 3.

**Table 3:** Echocardiographic findings (n = 55)

Variable	Median [p25 - p75]
LVEF Simpson	0,59 [0,5 - 0,65]
LA (mm)	39 [36 - 42]
LVFDD (mm)	51 [48 - 56]
LVFSD (mm)	32 [30 - 37,75]
Septum (mm)	10 [9 - 11]
Posterior wall (mm)	9 [9 - 11]
Mass index (g/m^2^)	124,5 [110 - 153,5]
PASP (mmHg)	32 [31 - 36]
Aorta root (mm)	34 [31 - 36]

LVEF: left ventricular ejection fraction; LA: measurement of the left atrium; LVFDD: left ventricle final diastolic diameter; LVFSD: left ventricular final systolic diameter; PASP: pulmonary artery systolic pressure.

In total, 50 patients completed the investigation with CC and five with CACT. In the first exam, three patients presented LMCA severe lesions (3.9%), 22 (28.2%) anterior descending coronary artery lesions (AD), 21 (26.9%) in right coronary artery, 2%) in circumflex coronary artery (CX). In patients submitted to CACT, one presented AD severe damage (1.3%) and one in RD (1.3%).

During hospitalization, 23 patients (29.5%) were submitted to intervention. Coronary transluminal angioplasty (CTA) was the main revascularization therapy. In three cases (3.8%), revascularization was surgical.

Comparing patients eligible for longitudinal strain analysis (group A) to those not eligible (group B), we found that group B had a lower proportion of women, a higher prevalence of diabetes, left cavities larger dimensions, larger root aorta diameter and lower systolic function on two-dimensional echocardiography; in addition to a higher ASA use rate, statins and beta-blockers, according to the data in Table 4.

**Table 4:** Clinical and echocardiographic characteristics of patients undergoing longitudinal strain analysis (group A, n = 15) compared to non-submitted patients (group B, n = 63)

	Group A		Group B		p
	Median	Interquartile Interval	Median	Interquartile Interval	
Age (years)	57	16	62	16	0,899
**Gender (%)**					
Male	33,3%		66,7%		0,037
Female	66,7%		33,3%		
BMI (Kg/m^2^)	28,62	7,2	28,12	6,56	0,903
SBP (mmHg)	143	33	137	31	0,510
HR (bpm)	78	23	74	14	0,824
Creatinine (mg/dL)	0,7	0,5	0,9	0,3	0,127
GRACE (points)	94	27	97	36	0,287
Hypertension (%)	80%		90,5%		0,363
Diabetes (%)	13,3%		44,4%		0,037
Smoking (%)	33,3%		31,7%		1
Dyslipidemia (%)	60%		66,7%		0,764
Family history for CAD (%)	26,7%		17,5%		0,470
LVEF Simpson	0,65	0,08	0,55	0,18	0,006
LA (mm)	37	5	40	9	0,009
LVFDD (mm)	48	5	53,5	9	0,007
LVFSD (mm)	31	6	37	7	0,112
Septum (mm)	10	2	10	3	0,668
Posterior wall (mm)	9	1	10	2	0,118
Mass index (g/m^2^)	109	49	133,5	26	0,095
PASP (mmHg)	34	13	29,5	10	0,895
Aorta root (mm)	31	4	35	4	0,006
**Medications in use (%)**					
ACEI	33,3%		31,7%		1
ARB	20%		46%		0,084
Beta blocker	20%		76,2%		< 0,001
Acetylsalicylic acid	60%		87,3%		0,023
Calcium channel blocker	26,7%		34,9%		0,762
Statin	53,3%		82,5%		0,035
Nitrate	40%		36,5%		1

BMI: body mass index; SBP: systolic blood pressure; HR: heart rate; LVEF: left ventricular ejection fraction; LA: measurement of the left atrium; LVFDD: left ventricular final diastolic diameter; LVFSD: left ventricular final systolic diameter; PASP: pulmonary artery systolic pressure. ACEI: angiotensinogen converting enzyme inhibitor; ARB: angiotensin receptor AT-2 blocker. Mann-Whitney was used for continuous variables (expressed in median and interquartile range) and Fisher's exact test for categorical variables (expressed as percentage).

Main causes to strain non-applicability were presence of prior infarction (56.4%), previous CTA (22.1%), prior surgical (CTA) revascularization (MRI), MRI and previous CTA (16, 7%), and presence of the following electrocardiographic alterations: LBBB, AF, pathological Q wave and pacemaker pace (12.8%).

In group A, patient majority presented low or intermediate risk, as detailed in Table 5.

**Table 5:** Risk score of patients submitted to longitudinal strain analysis

Score	Frequency (%)
**GRACE**	
≤ 108 points	86,7%
109-139 points	13,3%
≥ 140 points	0%

GRACE - Low risk - ≤ 108, moderate risk - 109 to 139, ≥ 140 - high risk

Coronary anatomy evaluation revealed a severe lesion in LMCA in 1 case (6.7%). Number of patients with severe lesions in coronary arteries AD, CX and RD was 2 (13.3%), 4 (26.7%) and 4 (26.7%), respectively.

**Figure 2:**
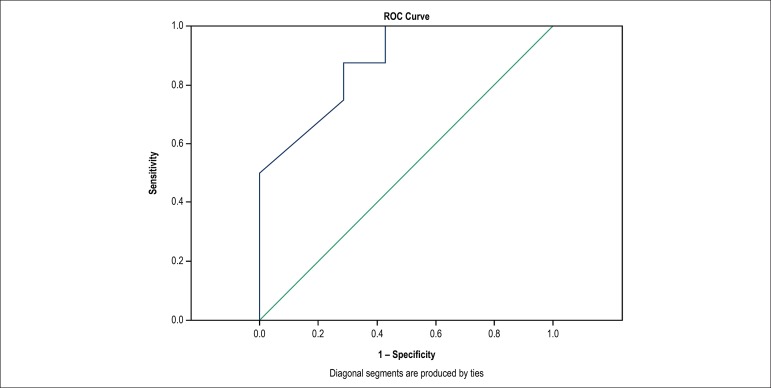
ROC curve to evaluate ability of global strain to identify severe lesion (> 70%) in any epicardial coronary artery. Area under the ROC curve 0.875, with p < 0.014.

**Figure 3:**
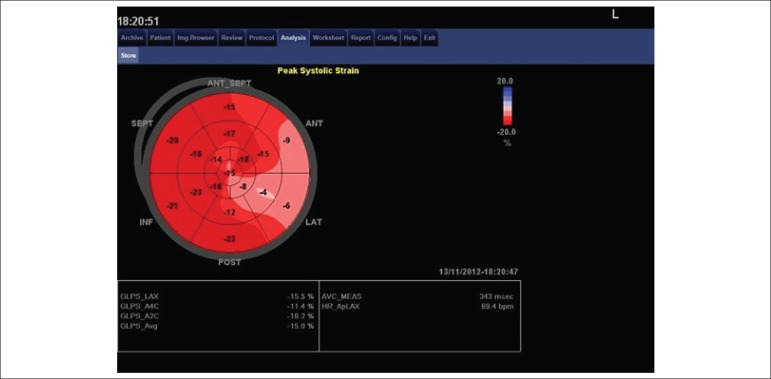
Case of patient with unstable angina, anterior descending coronary artery with 90% lesion in the proximal third and circumflex coronary artery with lesion of 70% in the middle third.

SL2D evaluation revealed a reduced global strain value in those who had severe lesion in any epicardial coronary artery (17.1 [3.1] versus 20.2 [6.7] with p = 0.014), area under the ROC curve 0.875, as shown in Figures 2 and 3.

Segmental strain assessment showed an association between severe CX lesion and longitudinal strain reduction of lateral wall basal segment (14 [5] versus 21 [10] with p = 0.04 and area under ROC curve = 0.864), and (12.5 [6] versus 19 [8] with p = 0.026 and area under ROC curve = 0.86).

## Discussion

Acquisition of images by ST with longitudinal strain determination allows a more complete myocardial function assessment and can detect subtle alterations in segmental contractility in ischemic heart disease patients, with good inter and intraobserver reproducibility.^7,12^ Thus, this method has been gaining more space in coronary artery disease evaluation, with a large number of studies produced in recent years.^15-17^

The present study is one of the pioneers in longitudinal strain applicability evaluation in UA patients attended at the Emergency Room of a Tertiary-level Cardiology Hospital.

The studied population clinical, electrocardiographic and echocardiographic characteristics demonstrate the complexity of patients with coronary artery disease (CAD). This probably justifies the method low applicability, since there are many variables (existing in the majority of patients studied) that could impair detection of reduced deformity due to ischemia. We emphasize that in the study population, 56.1% had previous infarction and 44.6% had previous cardiac procedure (CTA, MRI or both).

Shimoni et al.,^18^ evaluated SL2D in 97 hospitalized patients with angina and normal ventricular function; of these, 69 patients had major coronary disease. Global strain analysis was -17.3 ± 2.4 with an area under the ROC curve (AUC) of 0.80 to identify significant CAD in patients with angina; in the subgroup of patients with unstable angina the global strain also demonstrated good accuracy in predicting angiographic obstructive CAD (AUC = 0.86).^18^ Findings of this study are similar to those found in relation to strain diagnostic accuracy to identify significant CAD in angina , however, there was no reference as to method applicability to the sample.

We verified in the present study a statistically significant association between reduced global strain values and the presence of anatomically severe CAD, and similar accuracy to data available in the literature.^19^ When we analyzed segmental strain, we found a significant association only in basal segment deformity reduction of lateral and inferior walls, with stenosis ≥ 70% in CX and RD coronaries, respectively. We believe that segmental strain findings would be more robust if the sample was larger.

In a meta-analysis published in 2016 with 1385 patients included in 10 studies, global longitudinal strain demonstrated good accuracy in detecting moderate to severe CAD in symptomatic patients with AUC of 0.81, sensitivity of 74.4% and specificity of 72.1%.^19^

Despite the low SL2D applicability in ER and CU, most probably due to patients profile that our institution attends, current evidence and our findings indicate that this method may be a complementary exam in diagnostic algorithm of CAD and useful tool in early ischemia evaluation.

## Conclusion

In 80.8% of the cases, it was not possible to apply longitudinal strain, mainly due to the following criteria: presence of previous infarction or prior revascularization (percutaneous or surgical). We believe that the method applicability in a profile of patients with less clinical complexity would be greater, due to the method technical limitations.

In spite of this limitation, we can observe that the global strain showed a correlation with the presence of anatomically severe coronary lesion. In this way, SL2D could be included in the diagnostic arsenal of UA, in emergency units, since it is a noninvasive examination with diagnostic information available in a short period.
